# Raised SPINK1 levels play a role in angiogenesis and the transendothelial migration of ALL cells

**DOI:** 10.1038/s41598-022-06946-6

**Published:** 2022-02-22

**Authors:** Dong Luo, Dongqiang Liu, Chunbao Rao, Shanshan Shi, Xiaomei Zeng, Sha Liu, Hua Jiang, Lishi Liu, Zhenhong Zhang, Xiaomei Lu

**Affiliations:** 1 Medical Laboratory, Dongguan Children’s Hospital Affiliated to Guangdong Medical University, Dongguan City, Guangdong Province China; 2 Department of Medical and Molecular Genetics, Dongguan Institute of Pediatrics, Dongguan, Guangdong China; 3 Department of Hematology, Dongguan Children’s Hospital Affiliated to Guangdong Medical University, Dongguan City, Guangdong Province China; 4 Department of Neurology, Dongguan Children’s Hospital Affiliated to Guangdong Medical University, Dongguan, Guangdong China; 5 Department of Hematology-Oncology, Guangzhou Women and Children’s Medical Center, Guangzhou, Guangdong China

**Keywords:** Cancer, Cell biology, Molecular biology

## Abstract

The present study was designed to assess whether raised Serine protease inhibitor Kazal type 1 (SPINK1) expressions modulates angiogenesis. Human umbilical vein endothelial cells (HUVECs) exposed to SPINK1 were noted to exhibit raised expressions of interleukin-8 (IL-8) as well as VCAM-1 and ICAM-1 cell adhesion molecules in a dose-dependent manner. In co-culture system of HUVECs and Acute lymphoblastic leukemia (ALL) cells, SPINK1 exposure also resulted in enhanced endothelial cell motility and ALL cells trans-endothelial migration. High concentrations of SPINK1 caused in vitro cellular reorganization into tubes in Matrigel-cultured HUVECs and induced in vivo vascularization and brain infiltration of NOD/SCID ALL model mice. The further transcriptomic analysis indicated that SPINK1 treatment altered several biological processes of endothelial cells and led to activation of the MAPK pathway. This study is the first to determine the neovascularization effects of raised SPINK1.

## Introduction

Serine protease inhibitor Kazal type 1 (SPINK1) is also known as pancreatic secretory trypsin inhibitor (PSTI) or tumor-associated trypsin inhibitor (TATI). SPINK1 was previously thought to be mainly responsible for inhibiting premature trypsin activation in the pancreas^1^. Recent studies have demonstrated that SPINK1 is also performed other functions and highly expressed in several solid tumors, hinting towards its oncogenic potential^2^. Among human breast and colon cancers, SPINK1 is considered to be a transforming factor involved in the invasion and metastasis potential of cancer cells^3–5^, and SPINK1 contained in human colostrum has been shown to promote the proliferation and migration of HT29 cells^6^. Angiogenesis is an important event in tumor growth and hematogenous metastasis^7^. However, the possible contribution of SPINK1 in the molecular and cellular mechanisms of angiogenesis remains unclear, especially in hematological tumors, and there were growing evidence has highlighted the critical role of angiogenesis in developing and progressing ALL^8–10^. SPINK1 has been shown to interact with endothelial cells^11,12^. Recent research evidence shows that when mapping the transcriptome gene expression profiles of two prostate cancer cell lines mediated by SPINK1, significant expression of genes related to human endothelial cells (HUVEC) was observed^13^.

Based on these observations, this study seeks to investigate how elevated SPINK1 expression affects angiogenesis. Our results indicate that SPINK1 exposure to human umbilical vein endothelial cells (HUVECs) exerts significant effects on multiple checkpoints in the angiogenesis cascade ranging from cell signaling, adhesion, motility and cytokine production. SPINK1 also stimulated angiogenesis in a NOD/SCID ALL model mouse assay, implying that SPINK1 is crucial in mediating ALL angiogenesis.

## Materials and methods

### Cell culture, reagents and treatments

HUVECs were obtained from ScienCell (Carlsbad, CA, USA) and cultured in endothelial cell medium (ECM) in accordance to protocols stipulated by the supplier. RPMI-1640 medium supplemented with 10% fetal bovine serum was used to culture NALM-6 acute lymphoblastic leukemia cells. Sigma (St. Louis, MO, USA) was the sole supplier of all other experimental reagents unless otherwise indicated.

### Motility assays

Cell motility abilities were assessed using Transwell chemotaxis chambers (Corning Inc., Kennebunk, ME, USA)^14,15^ and wound repair assays. In brief, a total of 1 × 10^5^ HUVEC cells were cultured in upper chamber of a Transwell assay and allowed to form a monolayer. Nalm-6 cells (10^6^) were then added to the HUVEC monolayer and allowed to incubate for 6 h at 37 °C. This mixture of cells were then treated with chemoattractants containing SPINK1 (20 and 50 ng/ml) (R&D Systems, Minneapolis, MN), 10 ng/ml recombinant IL-8 (Sigma, St. Louis, MO, USA), or neutralizing antibodies against IL-8 (5 µg/ml) (Abcam, Cambridge, MA). The lower chamber of the Transwell assay contained 500 μl of RPMI 1640 medium supplemented with 10% FBS. The system was left alone for 6 h. The cells found in the lower layer were then fixed with 4% formaldehyde at room temperature for 10 min. Cells were rinsed, and additional cells in the upper chamber were wiped off. Cells were then stained with Giemsa. All cell lines underwent three separate experimental replicates, with the average number of migrating cells across three high-power fields per well quantified under 400 × magnification.

Confluent endothelial cell monolayers were gently scratched with a p1000 pipette tip for the wound healing assay. Cells were then rinsed with PBS. The scratched cellular monolayer was then incubated for 6 h with SPINK1-containing medium or control medium only. Cell-free images at various predetermined timepoints were then visualized with the Image-J and measured manually with the IMAGE-J software. The data is reported as the distance of cell migration in relation to those of the control cultures for each experiment.

### HUVEC tube formation on Matrigel

The in vitro effects of SPINK1 on vascular tube formation was tested using Matrigel as previously described^16^. Matrigel was used to house HUVECs in endothelial cell media prior to the addition of SPINK1. The cells were allowed to incubate for 6 h before being evaluated using phase contrast microscopy. Images of the total length of the cables and the number of meshes were measured using IMAGE-J software.

### In vivo angiogenesis assay

All animal experimental protocols were performed with strict regard to the Guangdong Medical University Legislation for Animal Care and was also approved by the Animal Ethics Committee of Guangdong Medical University. All methods involving animals were performed in accordance with the relevant guidelines and regulations. This study is reported in accordance with ARRIVE guidelines (https://arriveguidelines.org). Four-week-old female NOD/SCID mice (GemPharmatech Co., Ltd, Jiangsu, CN) were injected with 2 mg of cyclophosphamide (10 mg/ml) intraperitoneally two days before the injection of ALL cells. After 24 h, each mice received tail vein injections of either PBS (negative control) or Nalm-6 cells (5 × 10^6^ cells) at a concentration of 2.5 × 10^7^ cell/ml with or without 100 µg SPINK1. The mice were observed every 1–2 d, and changes in weight, coat color, activity, and appetite of the mice were recorded. The mice were sacrificed 15 d after injection. Femur samples and peripheral blood samples were taken for bone marrow and blood smears which were also stained with Wright Giemsa. Samples of liver, spleen, heart, brain, etc. were harvested and fixed with 40 g/L formaldehyde for 48 h. Paraffin blocks of the samples were made and sectioned in order for HE staining to be performed to observe the degree of tumor cell infiltration. Tibial sections and brain tissue sections were subjected to immunohistochemical staining (SP two-step method) to determine the presence of a huCD10 + CD19 + immunophenotype. CD34 is a marker of vascular lineage^13^, and bone marrow microvessel density (MVD) was calculated as previously described^8^. ‘Hot-spots’ on CD34-stained sections were identified at 200 × magnification. These areas represented areas of high vascularity. The number of microvessels were counted in 4 selected hot-spots at 400 × magnification. The MVD was determined to be the average total vessel count and was determined by two independent observers. Final MVD was taken to be the average count between the two observers. We did not note remarkable variability in results gained from the two observers.

### Statistical analysis

Data is depicted in terms of mean ± SEM based on the number of experimental repeats. An unpaired Student’s t-test was utilized to perform statistical analysis. *p* values of less than 0.05 were taken to indicate significant differences.

For more and detailed materials and methods, please refer to the supplementary materials.

## Results

### SPINK1 promotes endothelial cell proliferation and migration

SPINK1 has been shown to interact with endothelial cells to stimulate cell maintenance and growth ^11,12^. In a two-dimensional in vitro model, vascular endothelial cells which underwent a significantly long culture period were noted to spontaneously form a capillary-like network structure (CLS)^17^. HUVECs were found to crucially modulate the function of endothelial cells, vascular wall stretching, shear force and development response, as well as angiogenesis^18^.

**Figure 1:**
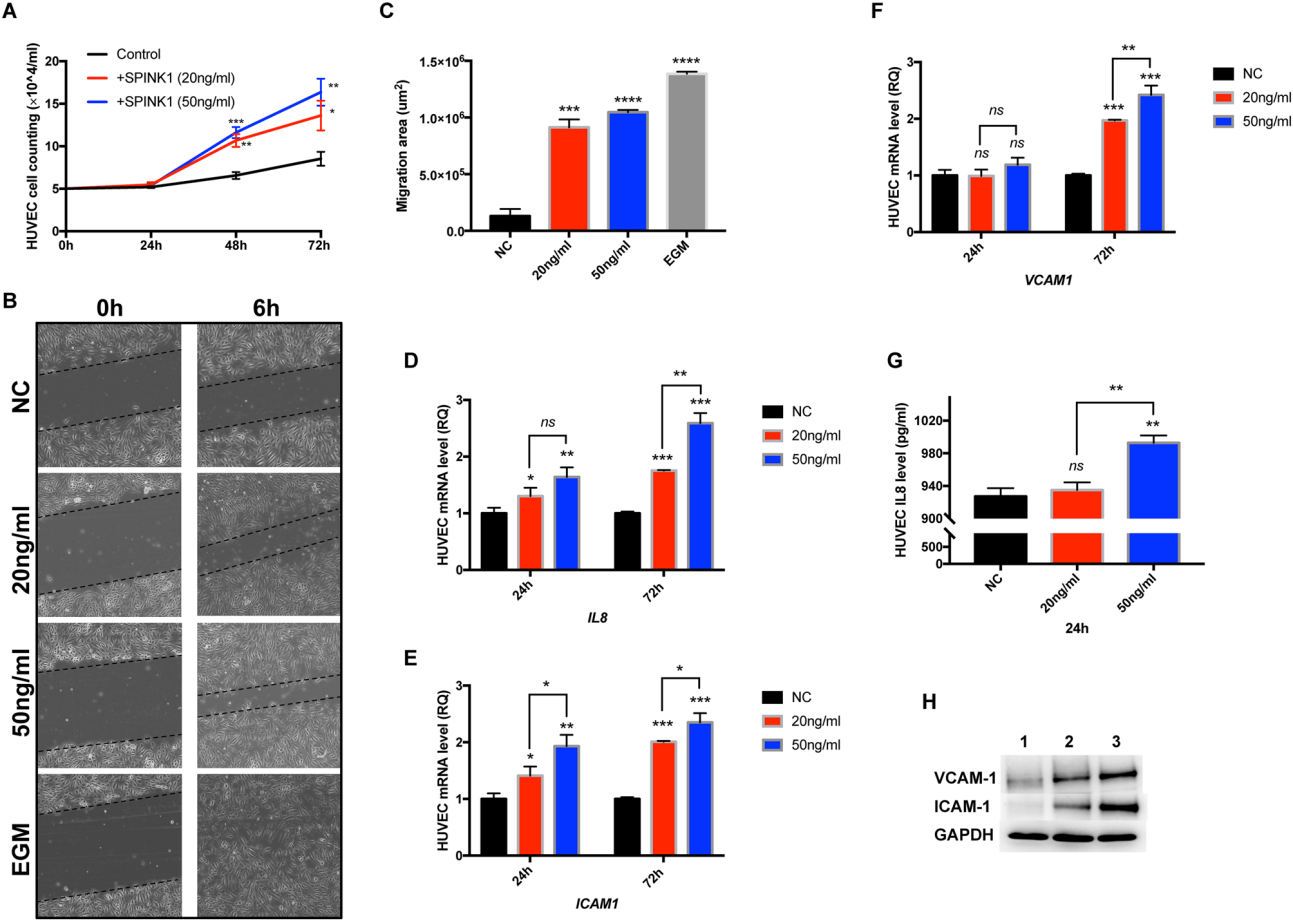
SPINK1 promotes endothelial cell proliferation and migration, and modulates IL-8, VCAM1, ICAM1 expressions in HUVECs. (**A**) Proliferation curve of endothelial cells generated upon various SPINK1 treatments (20 ng/ml or 50 ng/ml) in 72 h. Low-serum media was used as a negative control. (**B**) Endothelial cell monolayer with inflicted wound incubated with low serum medium (negative control), dose-dependent SPINK1 (20, 50 ng/ml), and endothelial growth medium (EGM, positive control) for 6 h. (**C**) Gap between wound edges assessed upon various SPINK1 treatments. (**D**–**F**) Various SPINK1 addition caused mRNA increase (IL-8, ICAM1 and VCAM1, respectively) in the endothelial cell monolayer. Low-serum media: negative control. (**G**) Measurement of IL-8 protein levels in HUVEC-CM were raised by SPINK1. (**H**) Immunoprecipitation and Western blot assay with anti-VCAM1 and anti-ICAM1 antibodies; HUVECs were cultured for 3 h with serum-free media followed by 72 h of incubation with 50 ng/ml SPINK1 (lane 2) or with low serum medium as negative control (lane 1) or with 10 ng/ml TNFα for 6 h as positive control (lane 3), stimulated ICAM1 and VCAM-1 protein expressions. The original images of all blots with three replicates are presented in Supplementary Fig. 10. Due to the blots were cut prior to hybridization with antibodies, the images of full-length blots were absent. *p ≤ 0.05, **p ≤ 0.01, ***p ≤ 0.001, ****p ≤ 0.0001; unpaired t-test.

Here, we once again proved that exposure of endothelial cells to different concentrations of SPINK1 can lead to significant cell proliferation in a time- and dose-dependent manner (Fig. 1A). Several physiological processes feature cellular migration, including angiogenesis. Endothelial cells were allowed to culture until a confluent monolayer was achieved before a wound was inflicted onto the surface. These wounded monolayers were exposed to different SPINK1 concentrations and subsequently assessed for degree of wound closure after 6 h. Endothelial cell migration was augmented by the presence of SPINK1 compared to the control media (Fig. 1B). In the positive control, endothelial growth medium (EGM)-treated cells migrated nearly completely close the wound after 6 h. Compared with the control, measurement of the wounded area in cells treated with 50 ng/ml SPINK1 found that it had healed by 75.5% (Fig. 1C).

### SPINK1 modulates IL-8 expression by HUVECs and induces cell–cell adhesion molecules

RT-PCR was used to quantify cytokine levels to identify if SPINK1 potentially induced genes responsible for mediating angiogenesis. IL-8, IL-6, VEGF, and TGF-β were selected as these cytokines have previously been found to be involved in angiogenesis and are also produced by endothelial cells upon exposure to various stimuli^19^. Figure 1D demonstrates that in contrast to the negative control, HUVEC monolayers exposed to SPINK1 resulted in a time- and dose-dependent increase in IL-8 mRNA levels in a statistically significant manner. Similar findings of raised IL-8 protein levels were also noted in HUVEC-CM exposed to SPINK1 (Fig. 1G). These findings were not found for IL-6, VEGF or TGF-β (data not shown).

The inflammatory process releases cytokines which go on to induce endothelial cells to secrete VCAM-1 and ICAM-1^20^. Varying concentrations of SPINK1 or control substrate were used to treat HUVECs to determine if SPINK1 affects mRNA expression of proteins responsible for cell–cell adhesion. Figure 1E,F demonstrates that the endothelial monolayer demonstrated time- and dose-dependent raises in ICAM-1 and VCAM-1 mRNA expression upon SPINK1 exposure (*p* ≤ 0.01). HUVECs treated with either SPINK1 or TNF-α (used as a positive control), subsequently displayed elevated ICAM1 and VCAM-1 protein levels (Fig. 1H).

### Stimulated ALL cells found to bind to the HUVEC monolayer

**Figure 2:**
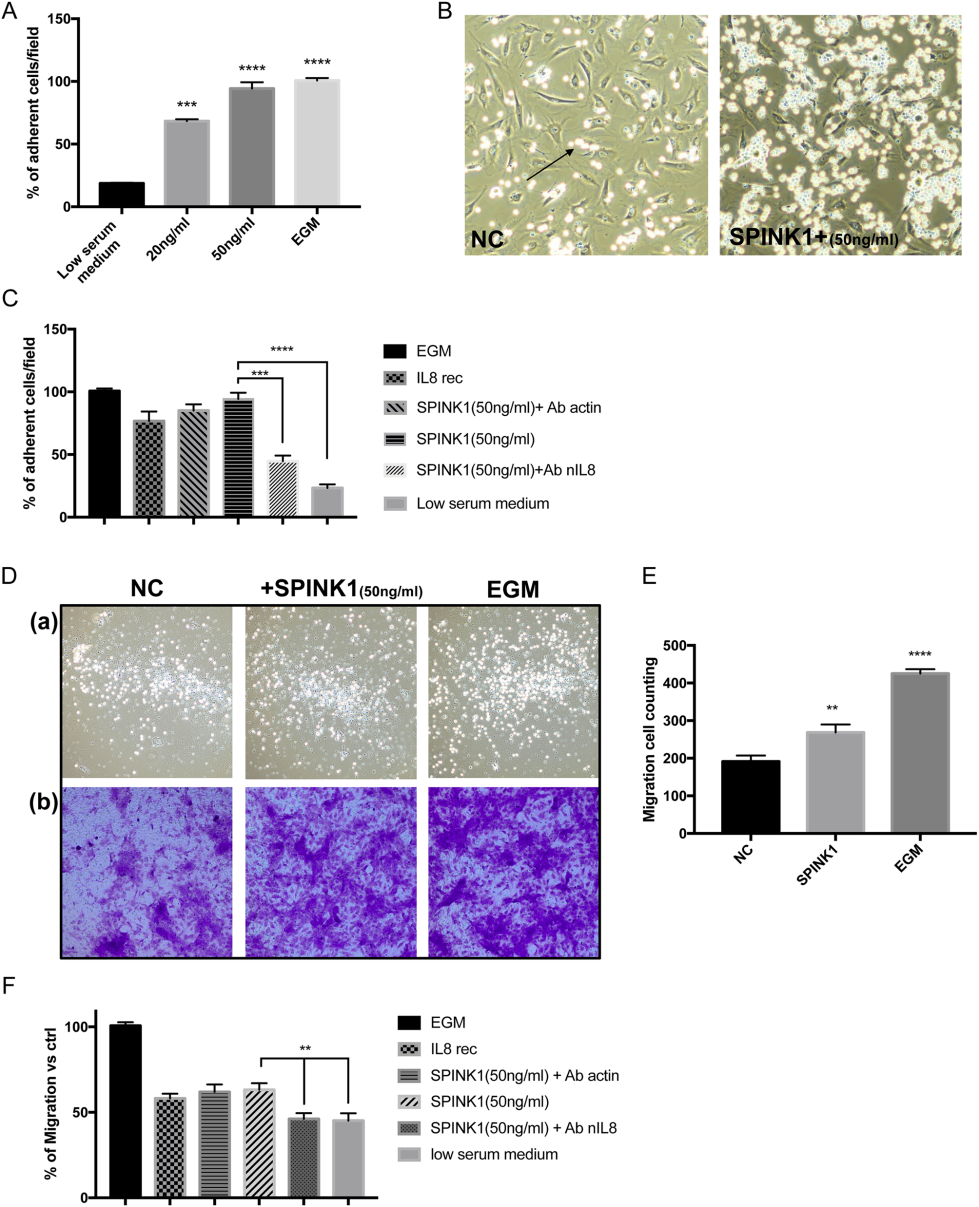
SPINK1 promotes ALL cell adhesion to HUVEC monolayers and trans-endothelial migration. (**A**) Endothelial cell monolayers were found to adhere to NALM-6 cells after a 6 h treatment period with various concentrations of SPINK1 or EGM as a positive control. (**B**) Cells (arrows) exposed to treatments as in A and imaged using phase contrast microscopy. (**C**) NALM-6 cells adhered to HUVECs exposed to 50 ng/ml SPINK1, 50 ng/ml SPINK1 plus anti-actin antibodies (5 µg/ml), 10 ng/ml recombinant IL-8, EGM (as a positive control), 50 ng/ml SPINK1 plus anti-IL-8 neutralizing antibodies (5 µg/ml), and low serum medium (as a negative control). (**D**) A Transwell system was used to assess the effects of SPINK1 on cell migration. The addition of 50 ng/ml SPINK1 or EGM as the positive control to the upper wells of the chamber caused an enhanced ALL cell migration (a) and HUVEC migration (b) after 6 h; low serum medium was used as the negative control (NC). (**E**) ALL cell counting in the lower wells of the chamber after 6 h. Values are the mean ± SD of 3 fields in three independent experiments. (**F**) Cell migration assay by Transwell coculture system. SPINK1 (50 ng/ml), SPINK1 (50 ng/ml) plus anti-actin (5 µg/ml), recombinant IL-8 (10 ng/ml), EGM (as a positive control), SPINK1 (50 ng/ml) in addition to anti- IL-8 (5 µg/ml), and low-serum media (as a negative control) were added. *p ≤ 0.05, **p ≤ 0.01, ***p ≤ 0.001, ****p ≤ 0.0001; unpaired t-test.

In order to further verify the biological phenotype caused by the increased expression of cell adhesion factors, we introduced ALL cells (Nalm6) for co-culture with HUVEC. Leukemia progresses as its cells gain the ability to adhere endothelial cells, allowing extramedullary infiltration^19^. NALM-6 leukemia cells were then cultured with the endothelial monolayer to determine if their adhesive abilities were mediated by raised ICAM-1 and VCAM-1 expression in SPINK1-treated HUVECs. As depicted in Fig. 2A, NALM-6 cells were more adherent to HUVECs after being exposed for 6 h to the indicated treatment in a dose-dependent manner. Figure 2B demonstrates that exposure to 50 ng/ml SPINK1 increased NALM-6 cells (arrows) adhesion to HUVEC monolayers. Figure 2C demonstrates that NALM-6 cells were also increasingly adherent to the HUVEC monolayer after the addition of recombinant IL-8. In contrast, treatment of HUVECs with SPINK1 plus anti-IL-8 neutralizing antibodies did not increase the endothelial cell adhesion to NALM-6 cells.

### SPINK1 promotes HUVEC migration and trans-endothelial migration of ALL cells

A Transwell assay was used to further determine the impact of SPINK1 on cell migration based on established protocols (Supplementary Fig. 1)^14,15,21^. When tumor cells adhere to endothelial cells, the trans-endothelial migration program can be induced, thereby allowing extramedullary infiltration^15^. Figure 2D,E show that the presence of 50 ng/ml SPINK1 stimulated enhanced ALL cell migration after 6 h. When recombinant IL-8 was used in place of SPINK1, a similar, a statistically significant effect on the stimulation of endothelial cell migration was also observed (Fig. 2F). In contrast, the presence of anti-IL-8 neutralizing antibodies in the upper wells of the chamber did not increase the motility of leukemia cells (Fig. 2F).

### SPINK1 stimulates in vitro tube formation*, *in vivo vascularization and brain infiltration

**Figure 3:**
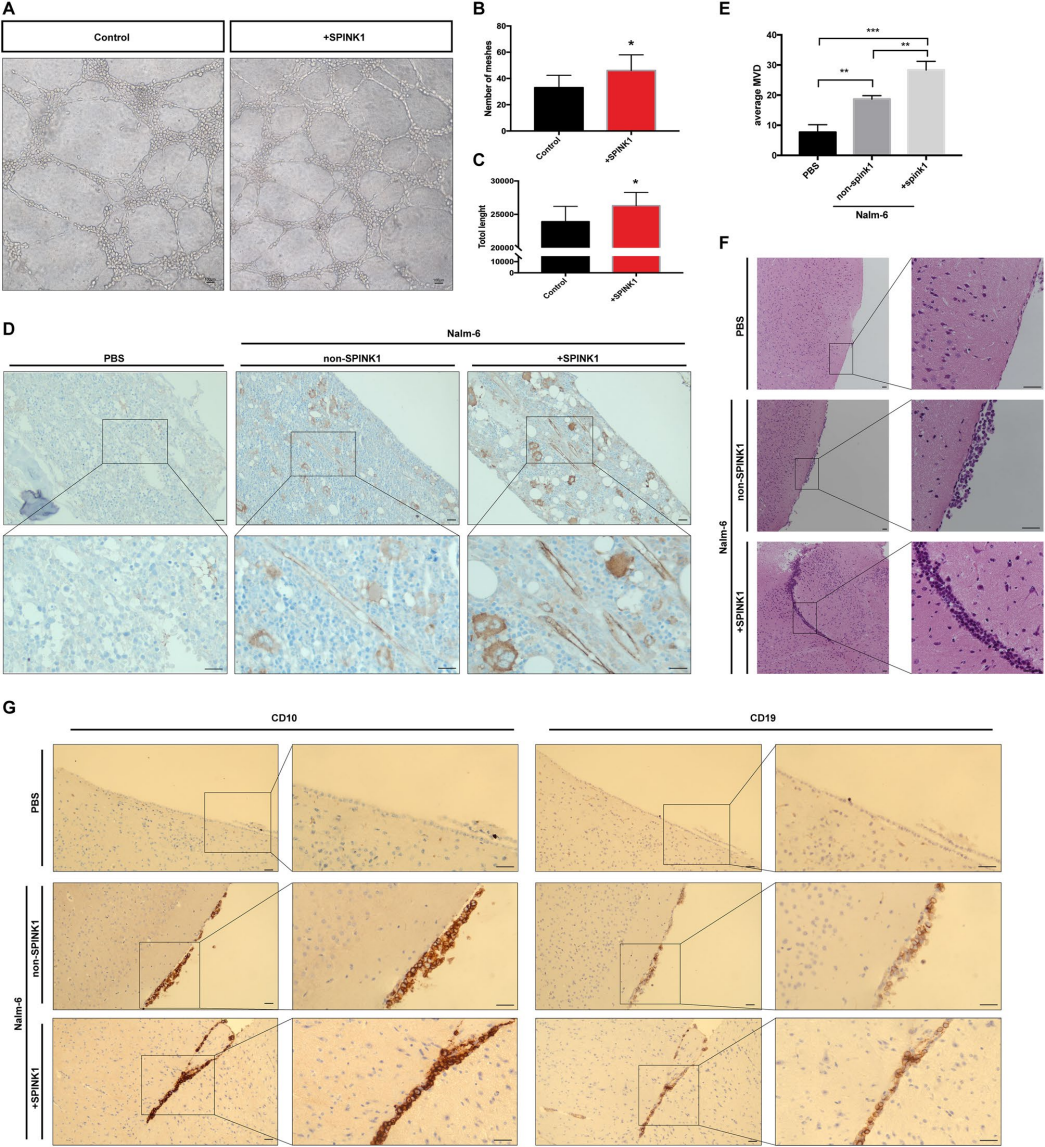
SPINK1 treatment stimulated in vitro and in vivo angiogenesis and brain infiltration. (**A**) Phase contrast micrographs demonstrating that SPINK1 induces endothelial network formation on Matrigel. Compared with the control group without SPINK1 treatment, adding 50 ng/ml SPINK1 to HUVECs enhanced formation of capillary-like structures (a marked raise in number of meshes and the total length of the cables). (**B**) Measurement of the number of meshes using ImageJ software. (**C**) Measurement of the total length of the cables using ImageJ software. (**D**) and (**E**) Average MVD calculation containing SPINK1 stimulates angiogenesis in B-ALL-NOD/SCID mice; tibial sections with immunohistochemical staining (SP two-step method) and CD34 as a marker of vascular lineage. **PBS**: Negative control, the normal NOD/SCID mouse with tail vein injection of PBS. **Nalm-6**: B-ALL-NOD/SCID model mice, the NOD/SCID mouse with tail vein injection of Nalm-6 cells (5 × 10^6^). **Non-spink1**: tail vein injection without SPINK1. + **SPINK1**: tail vein injection with 100 µg SPINK1. F: HE staining to be performed to observe the degree of tumor cell infiltration. G: The immunohistochemical staining to be performed to confirm the HE results. The NALM-6 NOD/SCID model mice treated with SPINK1 showed a deeper degree of brain tumor cell infiltration than that without the SPINK1 treatment. *p ≤ 0.05, **p ≤ 0.01, ***p ≤ 0.001, ****p ≤ 0.0001; unpaired t-test.

We established a model of angiogenesis using Matrigel-plated HUVECs in order to analyze potential cell differentiation into capillary-like structures^22^. Compared with the control group that was not treated with SPINK1, HUVECs treated with SPINK1 exhibited an increased cellularity with regards to network formation (Fig. 3A). The number of meshes (Fig. 3B) and total length of the cables (Fig. 3C) showed significant increases when treated with SPINK1. The angiogenic potential of SPINK1 was then assayed in vivo through bone marrow microvessel density (MVD) analysis of the B-ALL-NOD/SCID mouse model. Bone marrow smears (Supplementary Fig. 2) and immunophenotyping (Supplementary Fig. 3) demonstrated the effectiveness of the model. The analysis of the blood concentration of SPINK1 15 days after administration confirmed that vein-injected SPINK1 has a long-term effect in vivo (Supplementary Fig. 4). Figures 3D,E show that the bone marrow cavity of NALM-6 NOD/SCID model mice treated with SPINK1 was more vascularized than that without the SPINK1 treatment. The degree of bone marrow vascularization in ALL-NOD/SCID model mice was raised in contrast to the PBS control group. At the same time, the NALM-6 NOD/SCID model mice treated with SPINK1 showed obvious brain tumor cell infiltration, while most of the tumor cells of non-SPINK1 treated model mice were free of the pia mater (Fig. 3F). We used human CD10 and CD19 as ALL (Nalm6 cell line) markers^23^ and performed immunohistochemical staining on brain tissue sections to confirm the above results (Fig. 3G). No extramedullary infiltration of other tissues was found.

### SPINK1 treatment alters transcriptome-wide expression in endothelial cells

**Figure 4:**
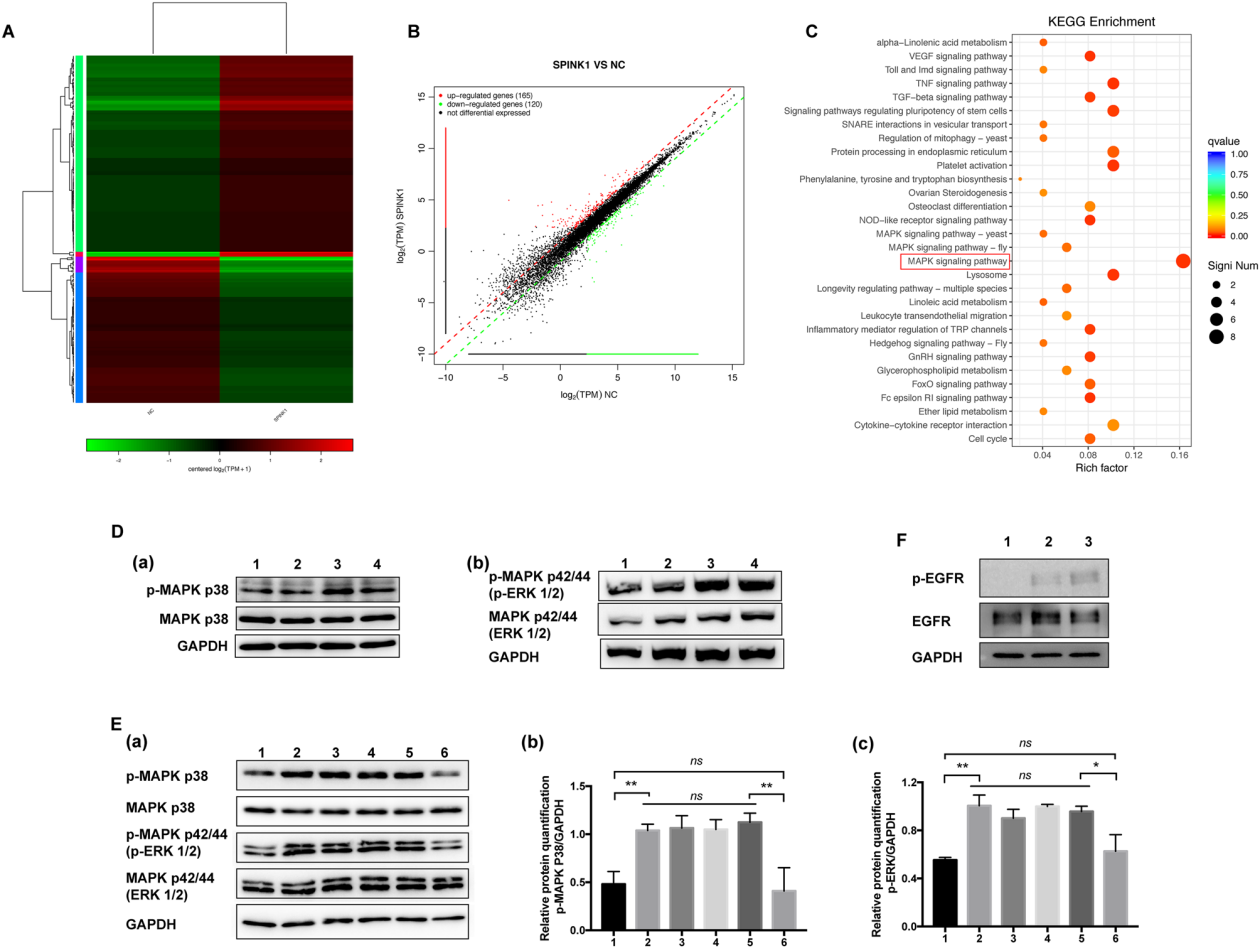
SPINK1 treatment alters transcriptome-wide expression of endothelial cells. (**A**) Unsupervised clustering based on differentially expressed genes (F test *p* ≤ 0.01). (**B**) Scatter plot representation of the comparison between low serum medium (NC)-treated and SPINK1-treated HUVECs. Genes up- or downregulated in SPINK1-treated cells relative to wild-type cells are shown in red and green, respectively. (**C**) Significantly enriched functional scatter plot (Kyoto Encyclopedia of Genes and Genomes, KEGG). (**D**) Western blot analysis of pMAPK and MAPK in HUVECs exposed to 50 ng/ml SPINK1. HUVECs were placed in serum-free media for 3 h prior to a 15 min exposure to 50 ng/ml SPINK1 (lane 3) and 30 min (lane 4) or with low serum medium alone for 15 min (lane 1) and 30 min (lane 2) as a control. (**E**) (a). IL8-dependent detection of MAPK phosphorylation. HUVECs were starved with serum-free media for 3 h, followed by 50 ng/ml SPINK1 treatment (lane 2), 50 ng/ml SPINK1 plus anti-actin antibodies (5 µg/ml) (lane3), 50 ng/ml SPINK1 plus anti-IL-8 neutralizing antibodies (5 µg/ml) (lane4), 50 ng/ml SPINK1 plus recombinant IL-8 (10 ng/ml) (lane5), 10 ng/ml recombinant IL-8 (lane6), or low serum medium (lane 1) treatment as a control. (b) and (c). The relative protein quantification of p-MAPK/GAPDH. (**F**) Western blot analysis of pEGFR and EGFR in HUVECs exposed to 50 ng/ml SPINK1. HUVECs were placed in serum-free media for 3 h prior to a 0 min exposure to 50 ng/ml SPINK1 (lane 1), 15 min (lane2) and 30 min (lane 3). Although quantitative comparisons between samples on different blots are unavoidable, the samples derive from the same experiment and that blots were processed in parallel. The original images of all blots with three replicates are presented in Supplementary Figs. 10 and 11. Due to the blots were cut prior to hybridization with antibodies, the images of full-length blots were absent. *p ≤ 0.05, **p ≤ 0.01, ***p ≤ 0.001, ****p ≤ 0.0001; unpaired t-test.

As our experiments thus far revealed the remarkable ability of SPINK1 to stimulate HUVECs, we further sought to uncover how SPINK1 might modulate the genetic expression profile of HUVECs. Transcriptome mRNA sequencing (mRNA-Seq) analysis revealed a total of 285 genes which were differentially expressed (twofold, *p* < 0.05) in HUVECs upon exposure to SPINK1 (Fig. 4A,B and Supplementary Table 1). These transcripts were mapped onto a gene ontology (GO) database, which revealed that the altered genes were involved specifically in biological adhesion, cell killing, cell aggregation, extracellular matrix component, receptor regulator activity, and chemoattractant activity (Supplementary Fig. 5). Further pathway enrichment analysis found both molecular functions and cellular components to be altered, specifically, the MAPK signaling pathway (p38 MAPK: MAPK13, MAPK 14; p42/44 MAPK: ERK1/2), which appeared to play a major role in these changes (Fig. 4C, Supplementary Fig. 6 and Supplementary Table 1). MAPK signaling pathway stimulation has been implicated in neovascularization ^24^. Based on this finding, we sought to determine if endothelial cells exposed to SPINK1 demonstrated MAPK pathway phosphorylation. As demonstrated in Fig. 4D, HUVECs exposed to 50 ng/ml SPINK1 demonstrated raised levels of phosphorylated MAPK-associated proteins (MAPK 14, ERK1/2), suggesting that SPINK1 may function to regulate HUVEC signal transduction. Furthermore, we used IL8 neutralizing antibody and human recombinant IL8 to intervene, and found that the phosphorylation of MAPK14 and ERK1/2 enhanced by SPINK1 was not IL8-dependent (Fig. 4E).

## Discussion

Angiogenesis is an important event in tumor growth and hematogenous metastasis^7^. More and more evidences show that SPINK1 is considered as a transforming factor in solid tumors and is involved in multiple stages of tumor progression, including invasion and metastasis^2^. However, the mechanism of SPINK1 in angiogenesis has not been well elucidated. As a matter of fact, SPINK1 has long been proven to act on endothelial cells to stimulate cell maintenance and growth^11,12^. Our study further finds that SPINK1 may enhanced neovascularization through the MAPK pathway.

This series of experiments demonstrate the impact of SPINK1 on in vitro and in vivo neovascularization. In fact, one of the first findings of our study was derived from a set of clinical data from two regions. We observed that elevated quantities of SPINK1 were present int the peripheral blood serum of children with ALL compared with healthy children (Supplementary Fig. 7A). Interestingly, compared with the extramedullary infiltration/relapse group and the complete remission group, the initial onset group had the highest level of SPINK1 and the complete remission group had the lowest level, which may suggest that SPINK1 is vital in mediating ALL progression. Latest research data suggests that ALL development and progression is strongly mediated by angiogenesis ^8–10^. Patients with ALL were noted to demonstrate marked neovascularization in their bone marrows along with augmented quantities of endothelial cells^8^. However, data is scarce regarding the exact molecular pathways by which the angiogenesis is induced in ALL.

The process of angiogenesis starts with endothelial cell activation, followed by cell adhesion molecules mediating intercellular interactions. Previous studies examining cellular adhesions demonstrate myoblasts adhere to cytokine-activated endothelium through ICAM-1 and VCAM-1 influences^25^. Endothelial cells treated with SPINK1 were found to secrete higher levels of ICAM1 and VCAM1 as well as demonstrated markedly increased cellular adhesion in a dose-dependent manner in contrast to untreated control cells.

Endothelial cells exposed to high quantities of SPINK1 may trigger endothelial activation and migration in the process of neovascularization. We find that SPINK1 induced the motility of HUVECs both in a Transwell chamber and in a wound healing assay. During endothelial cell migration or transendothelial migration of tumor cells, loss of cell–cell contact has been consistently observed^26,27^. Adding SPINK1 to endothelial cells may reduce intercellular adhesions, probably due to downregulation of zonule adhesion components such as VE-cadherin and β-catenin^19^. Increased cellular motility may be the result of suppressed local endothelial cell connections^28^. Other processes such as enhanced endothelial cell cytokine production and impaired vascular integrity may also lead to augmented tumor neovascularization. SPINK1 also stimulated increased protein and mRNA expressions of IL-8 in HUVECs. IL-8 belongs to the CXC chemokine family and functions as a potent proangiogenic^29^. Furthermore, we also found increased serum IL-8 in the peripheral blood of children with ALL and was consistent with the trend in SPINK1 (Supplementary Fig. 7B). The use of IL-8-neutralizing antibodies and recombinant IL-8 in our experiments proved that IL-8 was critical in mediating SPINK1-induced cell migration and adhesion. Interestingly, our data show that although the use of IL-8-neutralizing antibody will affect the adhesion and migration phenotypes, it will not drop to the background level, suggesting that SPINK1 itself may also have a direct promotion effect, which can be magnified by stimulating the production of IL8.

We employed both an in vitro Matrigel assay and an in vivo B-ALL-NOD/SCID model to evaluate SPINK1-triggered angiogenesis. Exposure to overloaded SPINK1 induced more abundant tubular differentiation of HUVECs and stimulated bone marrow cavity vascularization in B-ALL-NOD/SCID model mice. For the first time, raised SPINK1 in ALL was shown to be an important component leading to endothelial activation and angiogenesis. Furthermore, it is interesting that, similar to the in vitro ALL cell transendothelial migration experiment, high-dose SPINK1 seems to promote tumor cell infiltration in the brain tissue of ALL model mice.

A recent study showed that SPINK1 significantly altered prostate cancer cell genetic expression profiles, with two prostate cancer cell lines found to demonstrate an increase in gene expression related to human endothelial cells (HUVEC)^13^. Similarly, our research shows that SPINK1 reprograms the expression of the HUVEC transcriptome. GO analysis showed that the biological adhesion function of vascular endothelial cells mediated by SPINK1 changed significantly, which confirmed that the adhesion enhancement phenotype appears to be modulated by ICAM-1 and VCAM-1. Furthermore, there was a significant amount of chemoattractant activity which suggests that SPINK1 may act as both a chemokine and growth factor in angiogenesis. Based on further pathway enrichment analysis results, the MAPK signaling pathway was chosen for further scrutinization to determine if it was the link between SPINK1 and its angiogenic effect on endothelial cells. Endothelial cells exposed to angiogenic factors have been found to have increased levels of MAPK pathway activation^30^. MAPK signaling pathway has been proven to be a key player in neovascularization^24^. As predicted, SPINK1 was found to strongly activate MAPK in endothelial cells. In-depth mining of transcriptome data and in-depth molecular mechanism demonstration using MAPK signaling pathways as mechanism clues are the focus of our next work.

We try to explore the potential biological source of SPINK1 that stimulates vascular endothelial cells in vivo. We isolated exosomes from the peripheral blood serum of children with ALL, and we detected SPINK1. Specifically, the content of SPINK1 in the exosome portion was approximately half that in the non-exosome portion, and there were even cases where all SPINK1 was derived from the exosomes (Supplementary Fig. 8). This result illustrates the possible different sources of SPINK1 and implies that SPINK1 may have more than one receptor. The determination of the SPINK1 receptor has been controversial^31,32^. Ateeq et al.^33^ found that the cancer-promoting effect of SPINK1 as a growth factor has two pathways: EGFR-dependent and EGFR-independent, suggesting that SPINK1 may have different receptors on the cell membrane surface or in the cytoplasm. As Ateeq et al. described, we explored the SPINK1 receptor in HUVEC cell lines. Our results show that although only a low level of EGFR is expressed in HUVECs, SPINK1 stimulation still activates the autophosphorylation of EGFR (Fig. 4F). However, when we used the EGFR inhibitor gefitinib to treat HUVECs, SPINK1-mediated cell proliferation and migration were not significantly affected (data not shown), which may be the result of the EGFR-independent pathway, including the potential direct effects of the SPINK1 effect, Our data are insufficient to identify the SPINK1 EGFR-independent receptor or binding partner in vascular endothelial cells. However, we have shown that raised SPINK1 activates the MAPK signal transduction pathway in endothelial cells, leading to the release of activators of the angiogenic phenotype such as IL8. SPINK1 is over-expressed during the occurrence and development of ALL, which may not only activates endothelial cells to promote the angiogenesis process, but also promotes the transendothelial migration of ALL cells (Supplementary Fig. 9). Our findings suggest that novel ALL therapy may benefit from targeting SPINK1.

## Supplementary Information

Supplementary Information 1.

Supplementary Information 2.

Supplementary Information 3.

## Abbreviations

SPINK1: Serine protease inhibitor Kazal type 1
ALL: Acute lymphoblastic leukemia
HUVECs: Human umbilical vein endothelial cells
IL-6: Interleukin‐6
IL-8: Interleukin-8
MAPK: Mitogen-activated protein kinase
MVD: Microvessel density
NOD/SCID: Non-obese diabetic/server combined immune-deficiency
TGF‐β: Transforming growth factor‐β
TNF α: Tumor necrosis factor-α
VEGF: Vascular endothelial growth factor
VCAM-1: Vascular cell adhesion molecule-1
ICAM-1: Intercellular cell adhesion molecule-1
