# Supplementary material for: Raised SPINK1 levels play a role in angiogenesis and the transendothelial migration of ALL cells
Source: Sci Rep. 2022 Feb 22;12:2999. doi: 10.1038/s41598-022-06946-6 (PMC8864021; doi:10.1038/s41598-022-06946-6)
Supplement: Supplementary file 1 — Supplementary Information 1. [file 41598_2022_6946_MOESM1_ESM.pdf]

Raised SPINK1 levels play a role in angiogenesis and the transendothelial migration of ALL cells

Dong Luo¶, Dongqiang Liu¶, Chunbao Rao, Shanshan Shi, Xiaomei Zeng, Sha Liu, Hua Jiang, Lishi Liu, Zhenhong Zhang*, Xiaomei Lu*

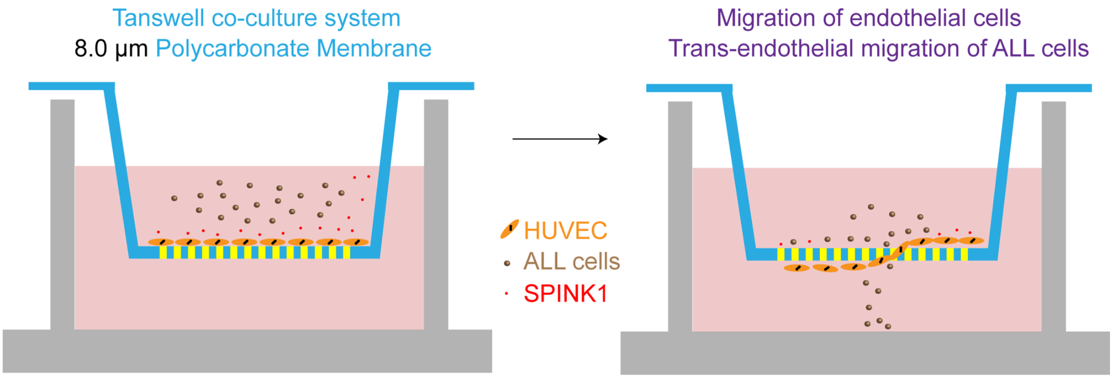

Supplementary Fig.1 Illustrative diagram of co-culture transwell system was used to assess the effects of SPINK1 on cell migration.

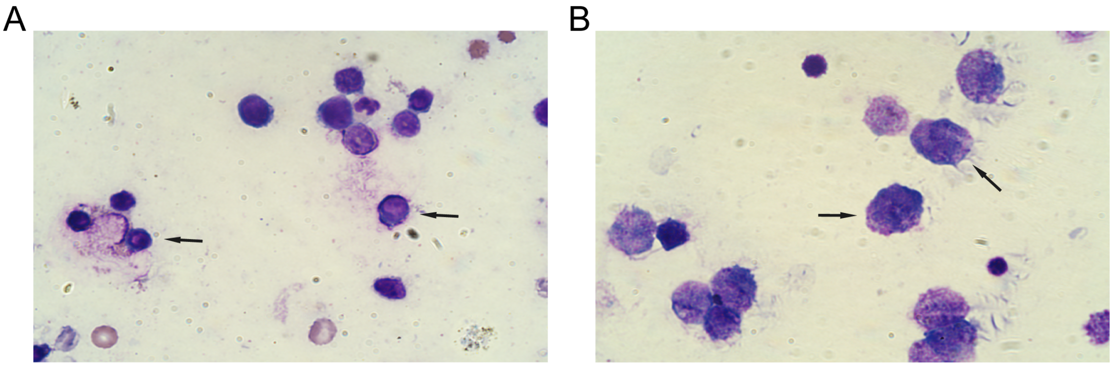

Supplementary Fig.2 Bone marrow smear of NOD/SCID mice after Wright Giemsa staining.

A: Bone marrow smear of normal NOD/SCID mice; the arrow indicates normal lymphocytes; B: Bone marrow smear of B-ALL-NOD/SCID mice; the arrow indicates leukemia cells (HE, ×1000).

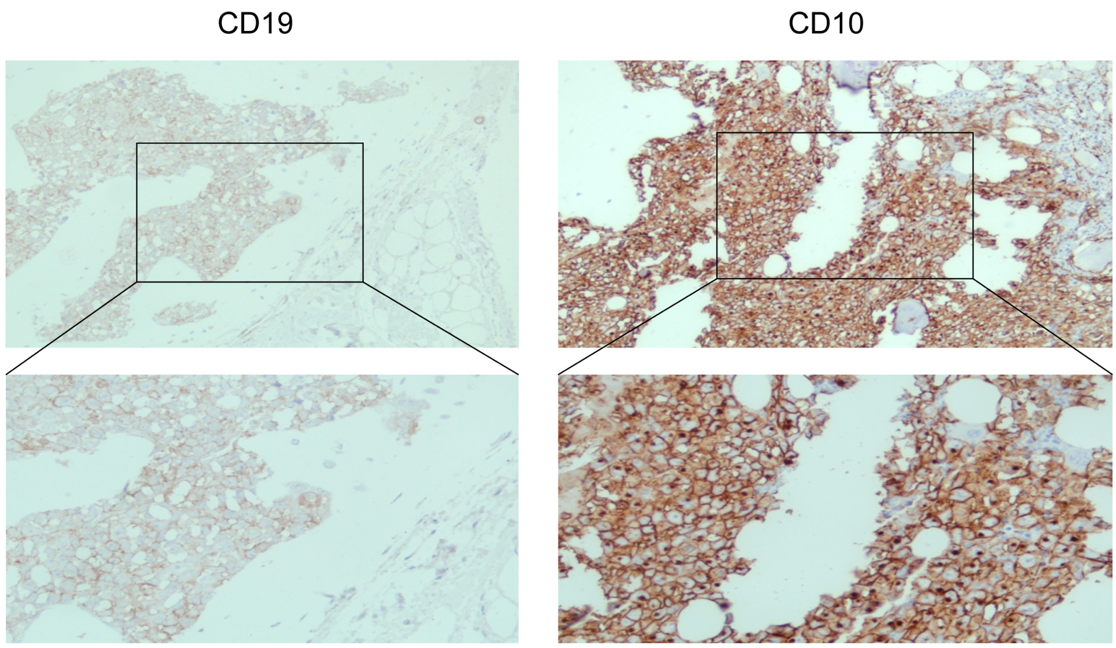

Supplementary Fig.3 Tibial sections with immunohistochemical staining (SP two-step method) to observe huCD10+CD19+ for immunophenotyping.

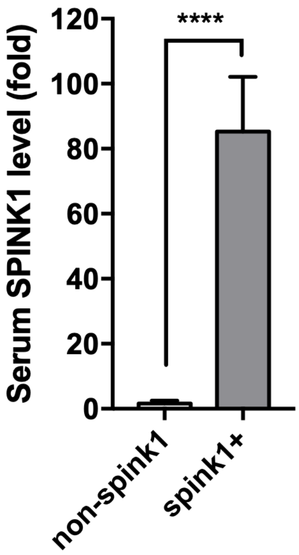

Supplementary Fig.4 The analyzation of blood concentration of SPINK1 15 days after administration.

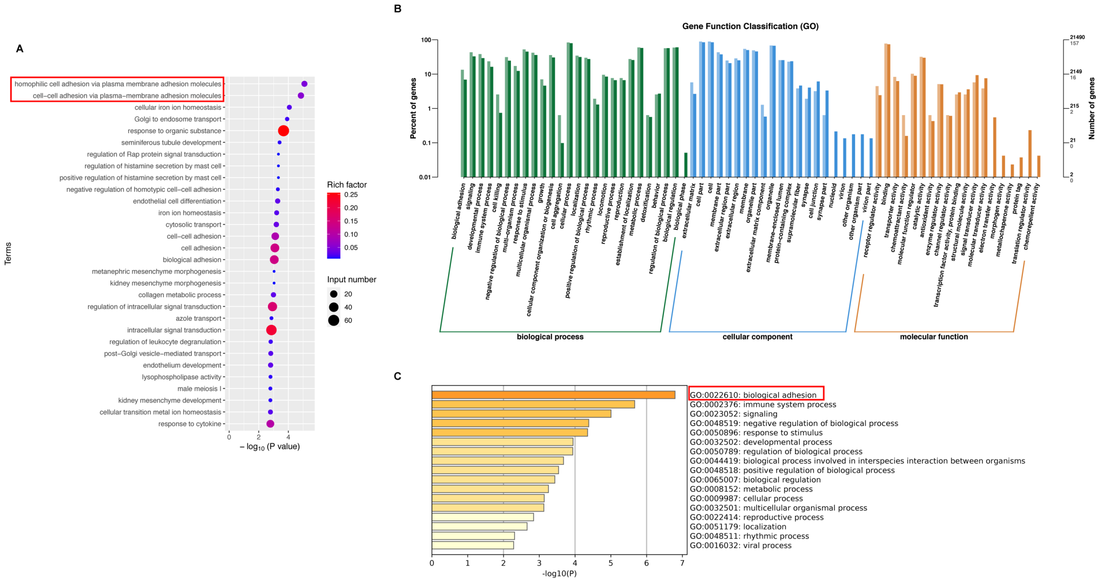

Supplementary Fig.5 GO analyses of differentially expressed genes between the SPINK1-treated and low-serum media (NC)-treated groups.

A. Significantly enriched function scatter plot. B. The histogram of GO annotation classification of differential genes. C. The GO analyses of differentially expressed genes from Metascape.

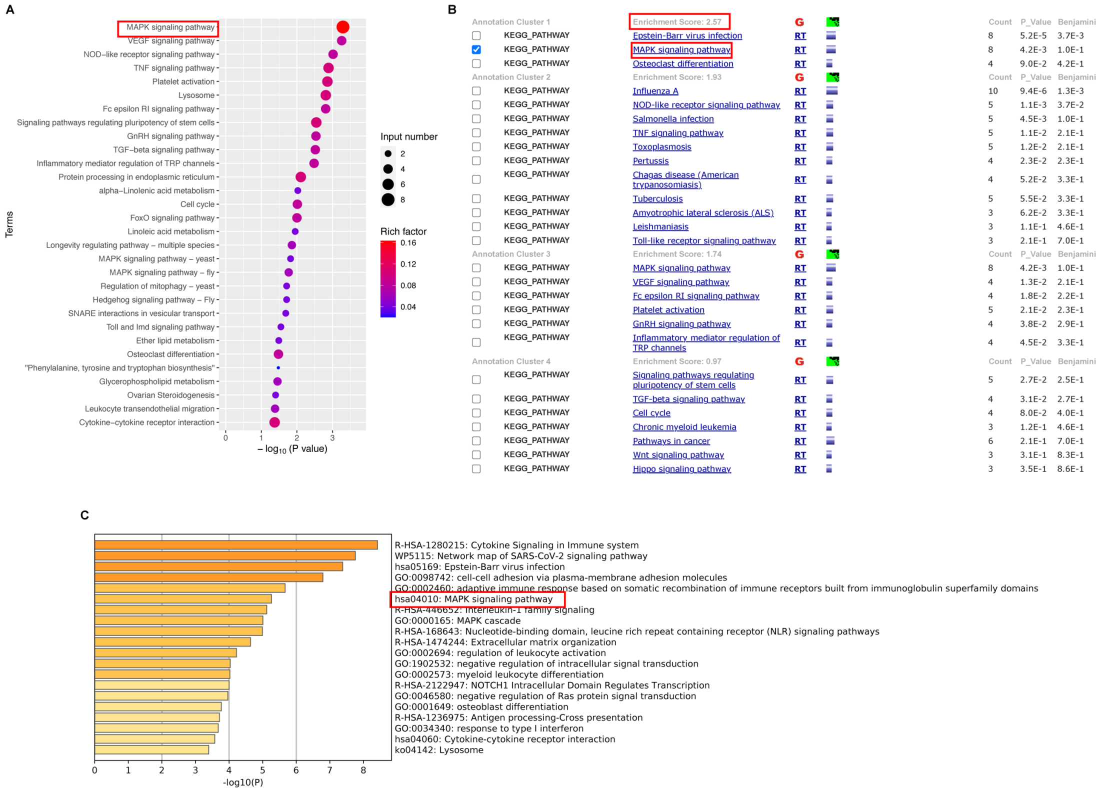

Supplementary Fig.6 KEGG pathway analyses of differentially expressed genes between the SPINK1-treated and low serum medium (NC)-treated groups.

A. The scatter plot of significantly enriched KEGG pathway. B. Significantly enriched KEGG pathway from DAVID database. C. The function and pathway analyses of differentially expressed genes from Metascape.

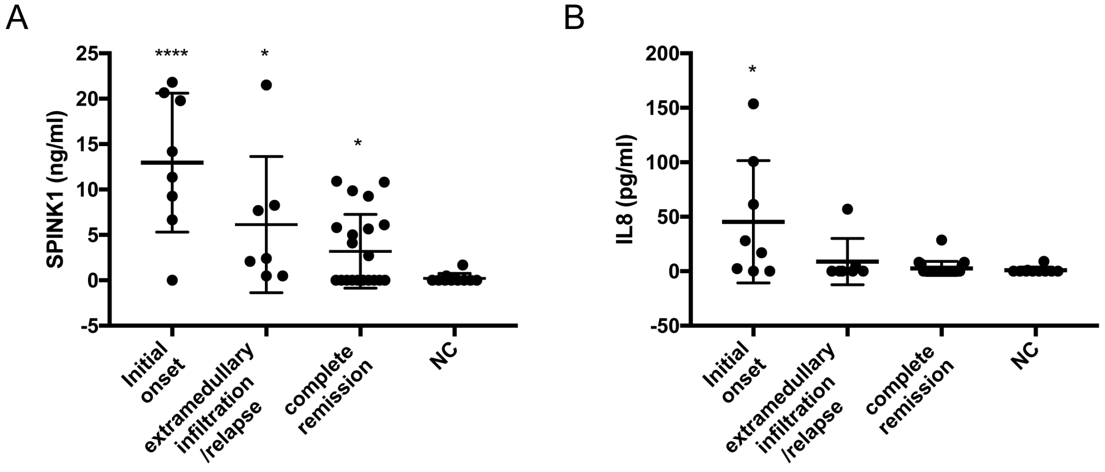

Supplementary Fig.7 Peripheral blood serum of children with ALL found to contain high expression of SPINK1.

A: Protein level of SPINK1 in the peripheral blood serum as quantified using ELISA (NC: healthy children group). B: Measurement of IL-8 protein levels in the peripheral blood serum of patients is consistent with the trend seen in SPINK1 expression.

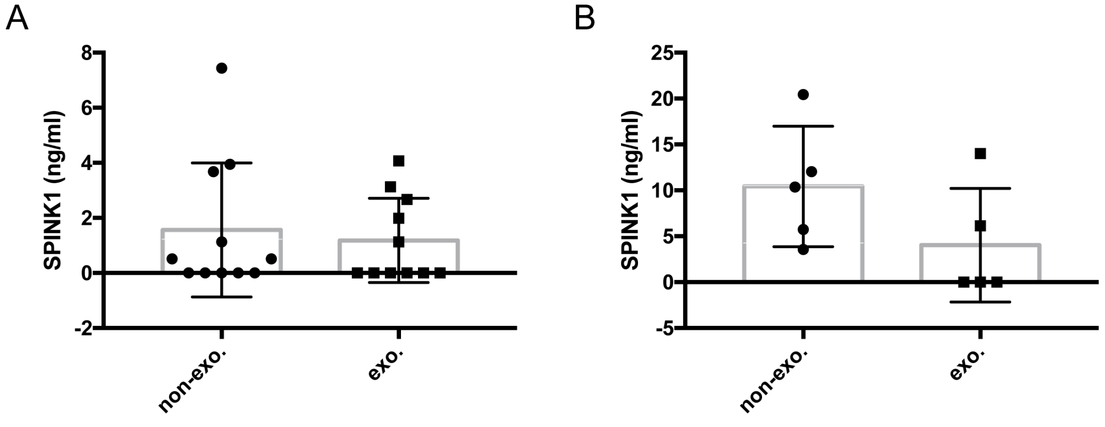

Supplementary Fig.8 The potential biological source of SPINK1 that stimulates vascular endothelial cells in vivo.

A: Exosomes were isolated from peripheral blood serum of ALL children in complete remission group, and then SPINK1 was detected. B: Exosomes were isolated from peripheral blood serum of ALL children in initial onset group and relapse group, and then SPINK1 was detected. Specifically, the content of SPINK1 in the exosome portion was approximately half that in the non-

exosome portion, and there were even cases where all SPINK1 was derived from the exosomes.

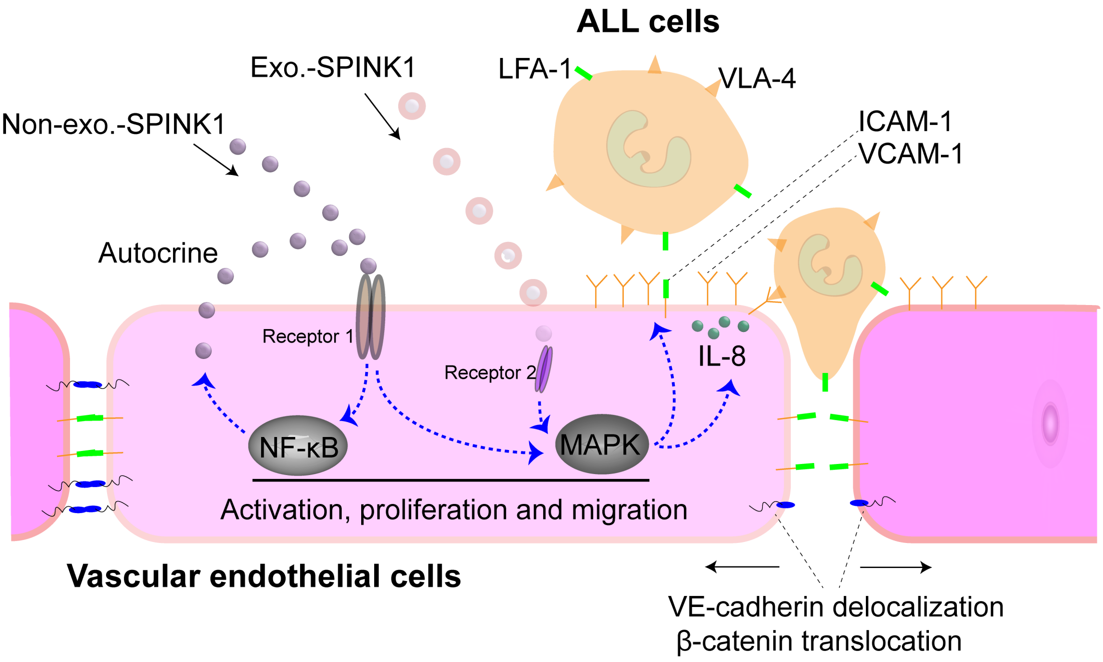

Supplementary Fig.9 Illustration of raised SPINK1's role in mediating angiogenesis and ALL cells trans-endothelial migration.

Pathological effects of SPINK1 from potential different sources on the signal network between ALL cells and vascular endothelial cells (HUVECs) and its potential as therapeutic targets and tumor biomarkers.

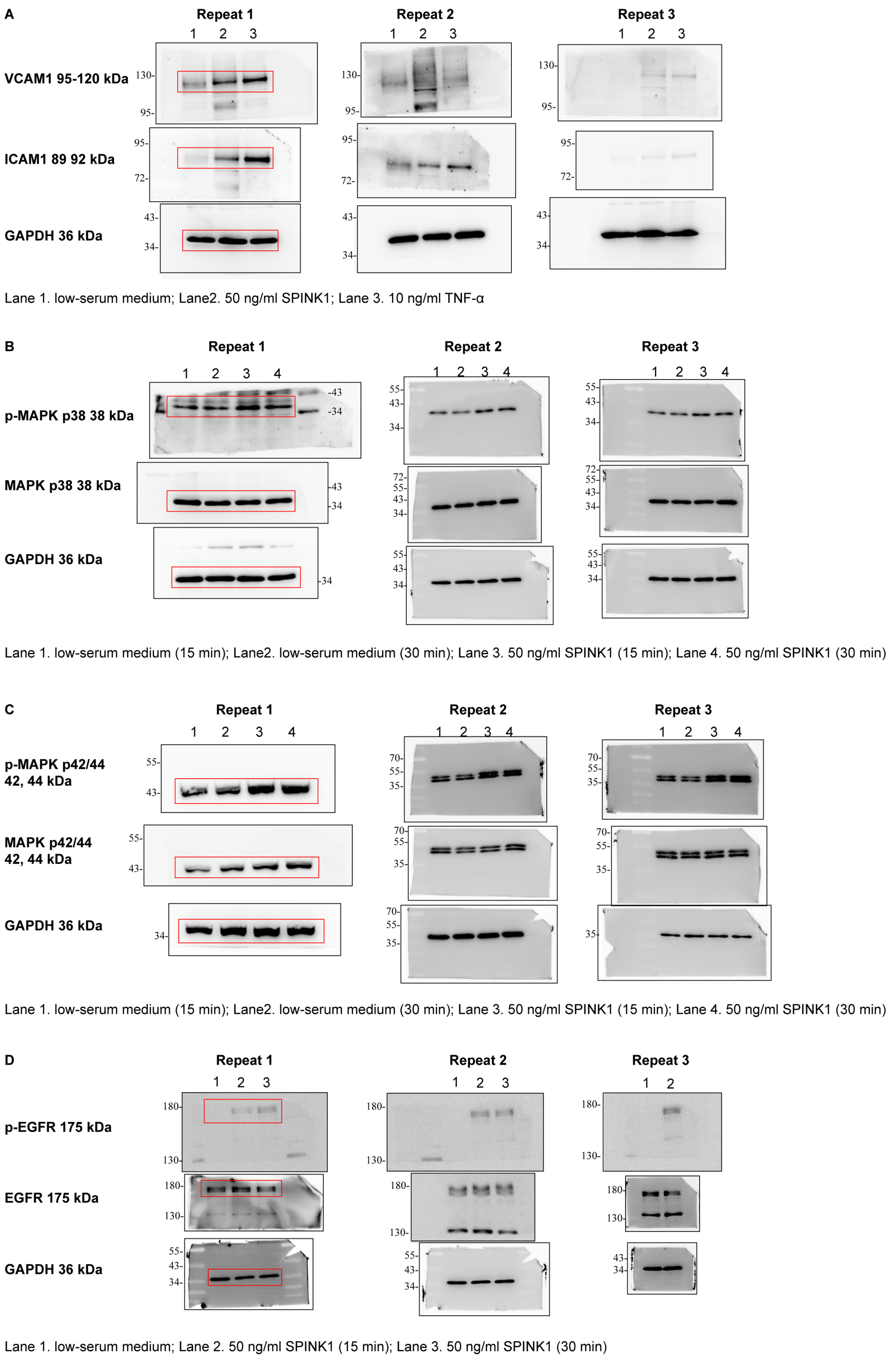

Supplementary Fig.10 The original images of all blots with three replicates corresponding to Figure 1H (A), Figure 4D (B and C), and Figure 4F (D).

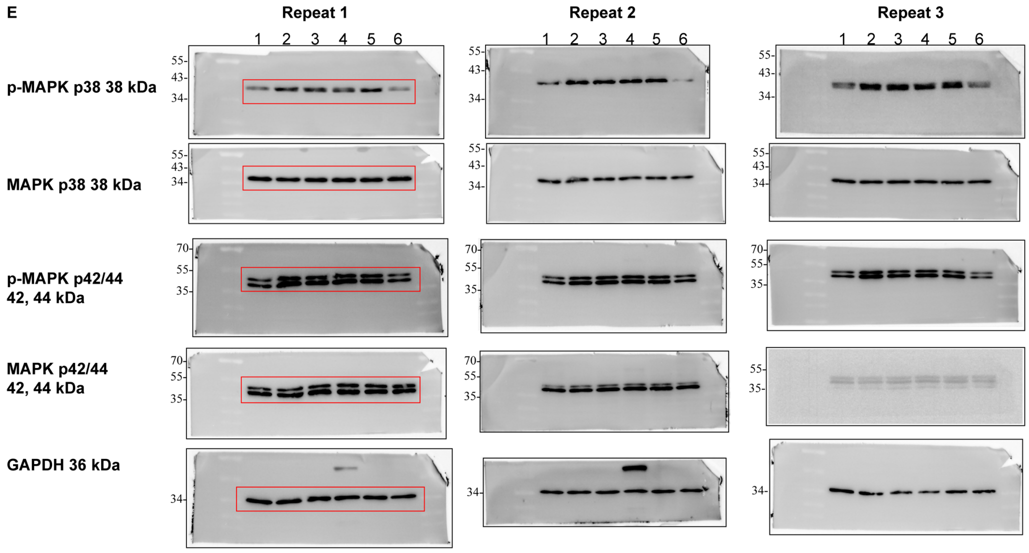

Lane 1. low-serum medium; Lane2. 50 ng/ml SPINK1; Lane 3. 50 ng/ml SPINK1 plus anti-actin antibodies (5 μg/ml); Lane 4. 50 ng/ml SPINK1 plus anti-IL-8 neutralizing antibodies (5 μg/ml); Lane5. 50 ng/ml SPINK1 plus recombinant IL-8 (10 ng/ml); Lane 6. 10 ng/ml recombinant IL-8.

Supplementary Fig.11 The original images of all blots with three replicates corresponding to Figure 4E(a).

Note: Due to the size of the table, it is provided as a separate file.

Supplementary Table 1 The annotation information table of gene expression difference analysis result between the SPINK1-treated and low serum medium (NC)-treated groups (6 hours). Genes of concern are marked yellow. (qValue<0.05, |FoldChange|>1.5, tpm>5)
