# Supplementary material for: Raised SPINK1 levels play a role in angiogenesis and the transendothelial migration of ALL cells
Source: Sci Rep. 2022 Feb 22;12:2999. doi: 10.1038/s41598-022-06946-6 (PMC8864021; doi:10.1038/s41598-022-06946-6)
Supplement: Supplementary file 2 — Supplementary Information 2. [file 41598_2022_6946_MOESM2_ESM.pdf]

Raised SPINK1 levels play a role in angiogenesis and the transendothelial migration of ALL cells

Dong Luo¶, Dongqiang Liu ¶, Chunbao Rao, Shanshan Shi, Xiaomei Zeng, Sha Liu, Hua Jiang, Lishi Liu, Zhenhong Zhang*, Xiaomei Lu*

Supplemental Material and Methods

Patients

Blood samples were extracted from 37 patients from two regions found to harbor B-lineage childhood ALL. These 37 individuals comprised of 8 initial onset patients, 7 extramedullary infiltration/relapse patients, 22 complete remission patients (average age 6.3± 2.4 years old), and 10 healthy children as a negative control (average age 6.2 ± 1.5 years old). The guardians of all children gave informed consent and all patients protocols were in accordance to the principles upheld by the Declaration of Helsinki and were approved by the hospital Ethics Committee.

ELISA

Whole blood samples were centrifuged for 15 min at 3000 rpm under room temperature. The resultant serum was subjected to SPINK1 protein concentrations quantification using an ELISA kit (R&D Systems, Minneapolis, MN, USA) based on protocols determined by the manufacturer.

HUVEC-conditioned media (CM) was harvested from cell cultures that had been exposed to the indicated treatment for 24 h. Cellular debris were removed by centrifuging CM aliquots, and IL-8 protein concentrations were measured with the same ELISA kit. Serum samples were also processed to measure IL-8 levels.

Exosome isolation and characterization

Briefly, Serum was isolated from the patient's whole blood sample. The serum underwent to a series of centrifugations (300 x g for 5 min; 2000 x g for 15 min and 10000 x g for 30 min) to sediment the cells and to remove cell debris. The supernatant was filtered 0.22 μm to eliminate vesicles with a size bigger than exosomes. Exosomes were then separated from the supernatant by centrifugation at 100 000 g x 90 min at 4 °C. The pelleted exosomes were washed with a large volume of PBS, re-centrifuged at 100 000 g x 90 min and finally resuspended in PBS for further analysis. The content of SPINK1 was measured in the exosome portion and the non-exosome portion respectively.

Cell proliferation assay

To generate the cell growth curve, 1×10^5 HUVECs were seeded in six-well plates to form the

HUVEC monolayers, then various SPINK1 added (20ng/ml and 50ng/ml), low-serum media was used as a negative control, and cell numbers were counted by Cell counter (BodBoge, Shenzhen, China) at 24, 48, and 72 hr. Each group was repeated at least three times.

Western blot and immunoprecipitation assays

The following antibodies were used in subsequent experiments: ICAM-1, VCAM-1 (Cell Signaling Technology, Beverly, MA), GAPDH, p38 MAPK, p42/44 MAPK, phospho-p42/44 MAPK, phospho-p38 MAPK (Santa Cruz Biotechnology, Santa Cruz, CA, USA), EGFR, and phospho-EGFR (Abcam, Boston, MA,USA). We followed the previous description (*Alessandro R, 2005*) with appropriate adjustments for ICAM1 and VCAM1 protein expression detection in HUVEC monolayers. 5 x 10^6 HUVECs were cultured for 3 hours with serum-free media followed by 72 hours of incubation with 50 ng/ml SPINK1 or with low serum medium (negative control) or with 10 ng/ml TNFα for 6 hours (positive control). Cell lysates were then subjected to immunoprecipitation experiments. An 8% SDS-PAGE gel was used to separate component proteins, which were then immunoblotted using anti-VCAM1 and anti-ICAM1 antibodies; The HUVECs were starved with serum-free media for 3 h, followed by 50 ng/ml SPINK1 treatment or low serum

medium treatment (both administered at 15 mins and 30 mins) as a control. In IL-8-mediated experiments, the HUVECs were starved with serum-free media for 3 h, followed by 50 ng/ml SPINK1 treatment, 50 ng/ml SPINK1 plus anti-actin antibodies (5 μg/ml), 50 ng/ml SPINK1 plus anti-IL-8 neutralizing antibodies (5 μg/ml), 50 ng/ml SPINK1 plus recombinant IL-8 (10 ng/ml), 10 ng/ml recombinant IL-8, or low serum medium treatment as a control. The samples were subjected to 8% SDS-PAGE electrophoreses, followed by immunoblotting with anti-p38, anti-p-p38 or anti-p42/44, anti-p-p42/44 antibodies; The HUVECs were starved with serum-free media for 3 h, followed by 50 ng/ml SPINK1 treatment (administered at 15 mins and 30 mins) or low serum medium treatment as a control. The samples were subjected to 8% SDS-PAGE electrophoreses, followed by immunoblotting with anti-EGFR, and anti-p-EGFR antibodies. Due to the blots were cut prior to hybridization with antibodies, the images of full-length blots were absent.

RNA extraction and real-time PCR

HUVECs were allowed to achieve a monolayer in 6-well plates before they were incubated for various times with different treatments. Levels of ICAM-1, VCAM-1 and IL-8 were evaluated using reverse transcription (RT) and TaqMan real-time quantitative polymerase chain reaction (RT-PCR).

The following primers were used: IL-8, forward 5’-GAAGTTTTTGAAGAGGGCTGAGA-3’, reverse 5’-TTTGCTTGAAGTTTCACTGGCA-3’; VCAM-1, forward 5’-TGTTTGCAGCTTCTCAAGCTTTTA-3’, reverse 5’-GTCACCTTCCCATTCAGTGGA-3’; ICAM-1, forward 5’-ATGGCAACGACTCCTTCTCG-3’, reverse 5’-GCCGGAAAGCTGTAGATGGT-3’; and GAPDH, forward 5’-TCGGAGTCAACGGATTTGGT-3’, reverse 5’-TTCCCGTTCTCAGCCTTGAC-3’ (Sangon Biotech, Shanghai City, China). All expressions were controlled against GAPDH.

Adhesion assay

Previously established protocols were used to carry out adhesion assays (Alessandro et al., 2005). The HUVEC monolayers were incubated for 6 h under the prespecified conditions. Cells were then rinsed with PBS before the addition of ALL cells for 1 h at 37°C. Three repeats were performed for each test and each cell condition contributed 5 high-power (400X) fields for quantification.

RNA-Seq and bioinformatics analyses

Gene library construction, sequencing, data preprocessing and gene mapping for the NC (negative

control, 1×10^6 HUVEC monolayers) and SPINK1 treated (50 ng/ml for 6 h) cell lines were performed by Sangon Biotech Co., Ltd. (Shanghai). The VAHTSTM mRNA-seq V2 Library Prep Kit for Illumina® was used to produced sequenced gene libraries, in compliance to instructions provided by the manufacturer. These libraries were then quantified and pooled, with the HiSeq X Ten sequencer (Illumina, San Diego, CA, USA) used to perform paired-end sequencing. FastQC (version 0.11.2) allowed us to assess the quality of the sequenced data.

StringTie (version 1.3.3b) was used to compute transcript gene expressions. A principal component analysis (PCA) and principal coordinate analysis (PCoA) were carried out to quantify the difference and distance between each experimental group. A |FoldChange| of more than 2 and a p value of less than 0.05 were indicative that a gene was significantly differentially expressed. Functional enrichment analyses including Kyoto Encyclopedia of Genes and Genomes (KEGG) and Gene Ontology (GO), were performed to discern differentially expressed genes (DEGs) which were significantly enriched in relative GO terms or corresponding metabolic pathways. The KEGG database is a public database comprising of several metabolic or signaling pathways, allowing for us to identify pathways which the identified DEGs are enriched in. GO is an international standard

classification system for gene function. Significant alteration was deemed when GO terms and KEGG pathways achieved a false discovery rate (q-value) of less than 0.05.
